# Controlling mildew of tobacco leaf by *Bacillus amyloliquefaciens* ZH-2 and its effect on storage quality of tobacco leaf

**DOI:** 10.1038/s41598-025-90058-4

**Published:** 2025-02-12

**Authors:** Hang Zhou, Yongfeng Yang, Tao Jia, Yangyang Yu, Siyuan Chen, Yao Qiu, Ruina Zhang, Hongli Chen

**Affiliations:** 1 College of Tobacco Science, Henan Agricultural University, Zhengzhou, 450046 China; 2 Technology Center, China Tobacco Henan Industrial Co., Ltd., Zhengzhou, 450000 China; 3 Tianchang International Co., Ltd., Baofeng Tobacco Redrying Factory, zhengzhou, 467400 China; 4 Deyang City Company Sichuan, Provincial Tobacco Company, Deyang, 618000 China

**Keywords:** Flue-cured tobacco, Storage, Fungi, *Bacillus*, Biocontrol, Quality, Plant sciences, Microbiology

## Abstract

Tobacco mildew is a common fungal disease that reduces tobacco quality, resulting in serious economic losses in the tobacco industry. In this study, the pathogens *Aspergillus niger*, *Aspergillus flavus*, and *Rhizopus arrhizus* were isolated from infected leaves. Furthermore, five plant endophytic bacteria isolated from healthy tobacco leaves were found to possess strong antifungal activity against these pathogens. Among these strains, *Bacillus amyloliquefaciens* ZH-2 exerted the strongest antagonistic effect against all mildew types (bacteriosphere diameter > 22 mm). The antagonistic action of ZH-2 was further observed using scanning electron microscopy, which revealed signs of contraction, deformation, and dissolution of the treated mycelia compared with that seen in the control group. The ZH-2 strain was found to produce high levels of proteases, chitinases, and β-1,3-glucanase, contributing to its antifungal activity via fungal cell wall rupture. The antifungal activity of ZH-2 was also demonstrated in the application test, as indicated by the significant reduction in mildew disease severity in tobacco leaves treated with this strain. Fermentation tests showed that the quality of ZH-2–treated, solid-state fermented tobacco leaves was superior to that of the control. Specifically, the alkaloid content significantly decreased by 10.62%, whereas the total and reduced sugar contents increased by 12.9 and 55.75%, respectively. Furthermore, macromolecular starch, cellulose, and protein contents significantly decreased by 25.85, 12.77, and 10.04%, respectively. These results indicate that the *Bacillus amyloliquefaciens* ZH-2 strain is effective against tobacco mildew and can improve tobacco quality upon solid-state fermentation.

## Introduction

Tobacco is one of the most important non-food agricultural products globally^1^. China is the world’s largest producer and consumer of tobacco, accounting for approximately one-third of the world’s annual tobacco consumption^2^. The storage conditions of tobacco leaves, particularly temperature, relative humidity, and the type of packaging materials used, can markedly impact the growth and development of mildew fungal pathogens. Tobacco leaves naturally contain a range of nutrients, including proteins and reducing sugars, which provide an ideal environment for mildew to thrive under certain storage conditions^3^. Therefore, tobacco leaves are susceptible to decay during storage, which can cause significant economic losses. Fermentation, also known as aging, is an important technique for improving tobacco quality^4^. Fermentation using microorganisms accelerates the process and enhances the substrate’s properties more effectively than natural fermentation^5^.

Mildew is filamentous fungi that are the main microorganisms causing mildew in tobacco^6^. To date, using chemical fungicides is the main method for controlling mildews; hoewever, their overuse has led to increasingly worrying resistance, pesticide residue, and environmental pollution problems^7^.Biological control using antagonistic microorganisms has proven to be a powerful tool for controlling fungal pathogens, and both its safety and environmental friendliness features have put it in the scientific spotlight^8–10^. Endophytes are microorganisms that colonize some or all of the life cycle of plant tissues or organs without causing adverse plant symptoms, including endophytes, bacteria, and actinomycetes. Endophytes control the pathogens, or secrete the secondary metabolites to inhibit the growth of the pathogens^11,12^. For example, an endophytic fungus isolated from Ocimum basilicum leaves was effective against the early eggplant disease *Aspergillus terreus*^13^. Similarly, Sneha et al. isolated endophytic antagonistic bacteria that inhibit pathogenic fungi from grape leaves, which can produce a variety of hydrolytic enzymes to inhibit fungal growth, thus playing a controlling role^14^.

Genera found in flue-cured tobacco causing mildew include *Aspergillus* and *Penicillium*, among others^15,16^. Pathogens responsible for tobacco mildew vary between regions. Welty^17^ reported that *Aspergillus stoloniferous* was detected in 81% of moldy tobacco samples collected in the United States. Tamayo^18^ the microorganisms causing moldy tobacco in Spain, mainly consist of *cocci*. Over the years, several chemicals have been utilized for tobacco leaf mildew management including fungicides such as chlorothalonil, carbendazim, and azoxystrobin, chemical elements such as acid phosphorus, manganese, and zinc; and natural antimicrobial compounds such as calcium salts and hydrogen peroxide^16^. Up to date, only a few studies related to the identification and biological control of mildew microorganisms during the storage period of tobacco in different geographic regions are available. Therefore, the development of alternative to chemical fungicides microbial control methods is crucial for the effective management of tobacco mildew diseases. Additionally, fermentation of tobacco leaves using microorganisms significantly improves their quality by modulating the internal chemical composition of tobacco, promoting the degradation of lignin, protein, starch and other biological macromolecules, and increasing the content of aromatic substances^19^. For example, both *B. subtilis* ZIM3 and its recombinant strain have been shown to exhibit high amylase and cellulase activities, leading to efficient biodegradation of starch and cellulose in tobacco^20^. However, to the best of our knowledge, there are no studies on the use of microorganisms for both mildew control and tobacco quality improvement.

In this study, fungal pathogens causing tobacco leaf mildew were isolated and identified using molecular techniques. A bacterial strain, selected after screening based on its antagonistic activity against these molds, was isolated from healthy tobacco leaves. The effect of the antagonistic strain on mold growth and tobacco leaf quality was further investigated by inoculating it on healthy tobacco leaves, providing a new strategy for simultaneously controlling flue-cured tobacco mildew and improving tobacco quality during storage.

## Materials and methods

### Tobacco leaves and chemicals

Rotten tobacco leaves (Yunyan 87) were collected from Changsha, Changde and Liuyang, Hunan Province, China, in 2022. Antagonist and pathogenic bacteria were grown on Luria-Broth (LB) medium and Potato Glucose Aar (PDA) medium, respectively, and kept in the laboratory.

### Isolation, screening, and pathogenicity testing of dominant mold pathogens

Tobacco leaves with mold symptoms (25 g) were weighed. Spores were collected via elution using 225 mL phosphate-buffered saline (PBS) to obtain a spore suspension. The suspension was oscillated at 28 °C at 180 rpm for 30 min. Serial dilutions of the spore suspension (10^−3^, 10^−4^, 10^−5^) were used to inoculate Bengal red agar medium plates using the plate pouring method, and incubated for 5 days at 28 °C. A large number of single colonies with different morphologies were selected and further purified separately. The purification process was repeated 2–3 times using the plate delineation method.

To test their pathogenicity, the isolated strains were inoculated on the surface of flue-cured tobacco sterilized at 121 ℃ for 20 min, and symptom severity was evaluated. Treatments 1–1 to 3, consisting of 10 mL from each strain (ZHM-1 to ZHM-3) sprayed on tobacco leaves were used to evaluate pathogenicity.Treatment with sterile water served as a control.

### Morphological identification

Morphological identification of pathogen strains from macroscopic and microscopic perspectives.The colony growth rate, colony morphology characteristics, sporulation, and spore structure were observed by microscope.Macroscopic morphological features included color and texture of colonies. Microscopic characteristics included spores as well as the morphology of the spore stem^21^.

### Molecular identification

Fungal genomic DNA was extracted using the fungal genomic DNA rapid extraction kit (Cargo number: B518229). The PCR products were amplified with the ITS universal primer ITS 1:5′-TCCGTAGGTGAACCTGCGG-3′ and ITS 4:5′-TCCTCCGCTTATTGATATGC-3′. The PCR reaction conditions included a pre-denaturation period of 3 min at 95 °C, followed by denaturation for 15 s at 95 °C, annealing for 15 s at 55 °C, extension for 2 min at 72 °C for 30 cycles, and finally an additional extension period for 5 min at 72 °C. Amplification products were sent to the Henan MENGKE Bio-technology Co. Ltd., China, for sequencing. The homology of the sequences was analyzed using the Basic Local Alignment Search Tool (BLAST) accessible on the NCBI Center for Biotechnology Information website. The phylogenetic tree was constructed using the Neighbor-Joining method with MEGA11.0 software and bootstrap values were calculated through 1000 replicates. Combined with the morphological observations, the taxonomic status of the bacterial strains was determined.

### Isolation and purification of antagonistic bacteria

Isolation of antagonistic endophytes was carried out by reference to the method of Hashem et al.^12^. Healthy tobacco leaves (15 g) were cut to appropriate sizes, transferred into a triangular bottle containing 85 mL of sterile water and shaken at 37 °C and 180 rpm for 5 h. Subsequently, leaves were transferred into 25 mL of LB at a 3% volume ratio and shaken at 37 °C and 180 rpm for 15 h. Appropriate serial dilutions were performed (10^−3^, 10^−4^, 10^−5^), and 100 µL of the diluted broth was spread on the surface of LB plates, and incubated at 28 °C for 16 h. Different colonies obtained from the original culture were inoculated on LB plates for purification using the plate scribing method and the procedure was repeated 2–3 times.

### Primary screening of antagonistic bacteria

The initial screening was performed using the plate confrontation method. Antagonistic strains were evaluated by a dual-culture bioassay^22,23^. Specifically, two parallel lines were drawn using the test fungus liquid 1.5 cm away from mold cakes placed in the center of a PDA plate, and each treatment was repeated three times. Plates were incubated in a constant-temperature incubator at 28 °C for 3 d. The presence or absence of bacterial inhibition was recorded.

### Rescreening of antagonistic bacteria

To further verify the antagonistic activity of endophytes against the isolated dominant molds, we screened by Oxford cup diffusion method with minor modifications referring to the method of Zhang et al.^1^. The Oxford cup was placed on a petri dish containing a layer of water agar. A volume of 10 mL of cooled to 40 °C PDA medium mixed with 100 µL of mold spore suspension (10^5^ CFU/mL) was poured into the Oxford cup. Subsequently, when the medium solidified, the Oxford cup was removed and agar was cut to create holes into the medium. Fermentation broth from the preliminary screening of the antagonistic bacteria was added into the agar holes. After 5 days of incubation at 28 °C, the diameter of the effective inhibition circle around the Oxford cup was measured using the crossover method, and each treatment was repeated three times. Strains with strong and broad-spectrum antimicrobial activity were selected for subsequent experiments.

### Molecular biological characterization of antagonistic bacteria

Genomic DNA of the antagonist bacteria was extracted using the Dynaeco (TSP701-50) Bacterial Extraction Kit and used as a template for PCR amplifications using the bacterial universal primers 27 F (5′-AGAGAGTTTGATCCTGGCTCAG-3′) and 1492R (5′-GGTTACCTTGTTAGACTT-3′).The 16 S rRNA region of antagonistic bacteria was amplified and sequencd, and the obtained sequences were analyzed via Basic Local Alignment Search Tool (BLAST) at the National Center for Biotechnology Information (NCBI).The phylogenetic tree was constructed using the Neighbor-Joining method with MEGA11.0 software and bootstrap values were calculated through 1000 replicates.

### Biochemical characteristics of isolated bacteria

The isolated bacteria were identified at a genus and species level by examination of their biochemical characteristics. The results were interpreted using Bergey’s Manual of Determinative Bacteriology^20^. A range of physiological and biochemical properties, including Gram staining test V-P determination test, citrate utilization test, nitrate reduction test, gelatin liquefaction test, methyl red test, starch hydrolysis test, hydrogen sulfide test and catalase test, were determined according to Lai et al.^24^.

### Observation of the effect of biocontrol bacteria on mycelial growth and morphology of pathogenic bacteria using scanning electron microscopy

The bacterial strain with the best antagonistic effect and pathogenic fungi were cultured on coverslips containing 2% water agar. After the strains matured, the coverslips were removed and fixed with 2.5% glutaraldehyde and kept for 1 d in a refrigerator at 4 °C. Subsequently, coverslips were washed with Phosphate Buffered Saline(PBS) solution three times (10 min each) and immersed in 1% osmium solution for ~ 2 h at 4 °C. After washing, samples were dehydrated in a series of 50, 75, 90, and 100% tert-butyl alcohol (10 min each), followed by gradient de-ethanolization with 50, 75, 90, and 100% tert-butyl alcohol for 10 min, respectively, drying at the critical point, and vacuum plating. The prepared fixed specimens were observed under a scanning electron microscope and the results were recorded. Three strains of the dominant mold pathogen grown for 5 d naturally, without any antagonist, were used as control.

### Determination of cell wall hydrolase activity of antagonistic bacteria

To examine the role of the cell wall–degrading extracellular enzymes secreted by antagonistic bacteria in suppressing the mycelial growth of the fungal pathogens, the enzymatic cell wall hydrolase activities were evaluated. A volume of 100 mL from the bacterial fermentation broth was collected and centrifuged for 10 min at 4 °C and 8000 rpm. The supernatant was discarded and 5 mL of 50 mmol/L KH_2_PO_4_–K_2_HPO_4_buffer solution with a pH of 7.5 was added to the remaining solution which was then ultrasonically crushed. Subsequently, the activities of chitinase was determined using specific assay kits. The activity of β −1,3 glucanases was determined by using the dinitrosalicylic acid (DNS) method^25^. Protease activity was determined using the Choi method^21^. One unit of enzyme activity is defined as the amount of enzyme that produces 1 µg of N-acetamido-glucose per 10,000 chitin-degrading bacteria per hour at 37 °C. The amount of enzyme that causes the production of 1 mg of reduced sugar per hour per 10,000 bacteria at 37 °C is defined as one unit of enzyme activity. The amount of enzyme that produces 1 µg of tyrosine per minute per 10,000 bacteria is defined as one unit of enzyme activity.

### Effect of antagonistic bacteria on moldy tobacco leaves

The leaf spray method was used to test the effect of strain W1 on mildew.First, the control effect of individual fungi was verified. After the 60 g tobacco leaves were sterilized, 10 mL of each mold spore suspension was sprayed. The control group was sprayed with mold spore suspension before 10 mL of antagonistic spore suspension.In 28℃ and 75% relative humidity conditions for 15 d, the mildew degree of tobacco leaves was observed, and the treatment was repeated three times.

Subsequently, the efficacy was verified against mixed fungi. After sterilization, 60 g of tobacco leaves were spray with mold mixed spore suspension and stationary for 30 min. Each group was sprayed with 10 mL of antagonistic suspension 10 mL (10^6^ CFU/mL, 10^7^ CFU/mL, 10^8^ CFU/mL, 10^9^CFU/mL) with 10 mL sterile distilled water as the control group. In 28℃, 75% relative humidity conditions for 20 d, and the treatment was repeated three times.Combined with the degree of tobacco mildew disease, scoring was performed using the condition grading criteria of Zhou et al.^26^ level 0, not infected; level 1, symptoms covering less than 5% of the total leaf area; level 3, symptoms covering 5 to 25% of the total area; level 5, symptoms covering 25 to 50% of the total leaf area; level 7, symptoms covering 50 to 75% of the total leaf area; and level 9, symptoms covering more than 75% of the total leaf area. The disease index was calculated based on the grading results, and the relative control effectiveness was calculated based on the disease index.

$$\text{Incidence rate} = \text{number of incidence samples}/\text{total number of samples} \times 100\%$$

$$\begin{aligned} {\text{Disease index}} & = \left[ {\sum {({\text{number of incidence samples}} \times {\text{corresponding number}}} } \right. \\ & \quad \left. {\text{of grades})/({\text{total number of samples}} \times {\text{highest disease grade}})} \right] \times 100 \\ \end{aligned}$$

$$\begin{aligned} {\text{Relative control effect}} & = \left[ {({\text{control disease index}} - {\text{treatment}}} \right. \\ & \quad \left. {{\text{disease index}})/{\text{control disease index}}} \right] \times 100\% \\ \end{aligned}$$

### Effect of antagonistic bacteria on tobacco leaves fermentation

The selected strains obtained from primary and secondary screening were formulated into a bacterial suspension containing 10^9^ CFU/mL and applied on the flue-cured tobacco using sprayers. Two treatment groups were prepared, and incubated for 7 d in a container under constant temperature (35 °C) and humidity (60%) conditions. The first treatment (2 − 1) consisted of 5 mL of water sprayed on 50 g of tobacco leaves, whereas the second (treatment 2–2) consisted of 5 mL of fermentation solution sprayed on 50 g of tobacco leaves.

### Determination of conventional chemical composition

According to the method of Yao et al.^28^, 0.25 g of dried tobacco powder is taken, 25 mL of extract (acetic acid and ethanol volume fractions of 1% and 2%, respectively) is added, shaken for 1 h and the filtered supernatant is determined with a continuous flow analyser.

### Determination of macromolecular substances

Sample pretreatment involves using a soft brush to clean the dust from the surface of the tobacco leaves, removing the main vein, cutting them into fine strands, drying them in a 60℃ oven, grinding them into powder, and passing them through a 60-mesh sieve for storage.The starch, cellulose and lignin, and protein contents were determined according to YC/T 216–2013, YC/T 347–2010, and YC/T 249–2008, respectively^29,30^.

### Volatile flavor compound analysis

The volatile flavor content was determined by a slightly modified Mai’s method^31^. The tobacco sample should be weighed in a 100 mL round-bottom flask at 20.00 g. Thereafter, the following substances should be added sequentially: 2.0 g of citric acid, 0.5 mL of internal standard, and 5 Next, 0.00 mL of deionized water was added, and the solution was mixed thoroughly. The organic phase was collected using a simultaneous distillation extraction apparatus with 40 mL of dichloromethane as the extracting agent, and the sample was concentrated to approximately 1 mL. Subsequently, the sample was analyzed using gas chromatography-mass spectrometry (GC/MS) with a 0.45 μm filter membrane in the sample vial.

### Statistical analysis

The software SPSS 27.0 (SPSS Inc., Chicago, IL, USA) was used for statistical analysis. One-way analysis of variance was performed, and the least-significant difference post hoc method at *P* < 0.05 was used for multiple sample comparisons. Independent sample t-test was applied to compare the means between the control and each test group at *P* < 0.05.

## Results

### Morphological identification and molecular biological identification of dominant mildew

**Fig. 1:**
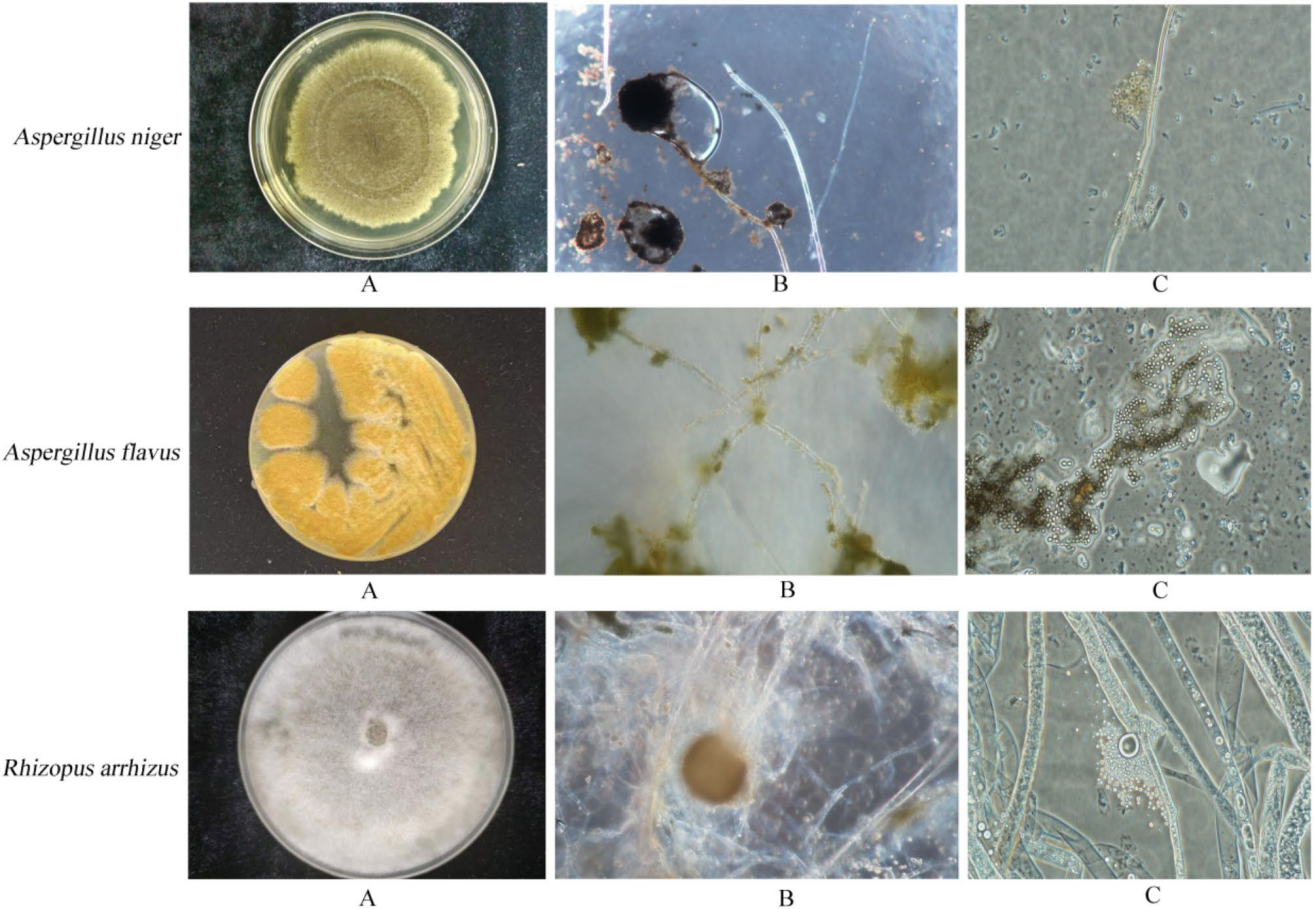
Colonies and microscopic images of strains ZHM-1, ZHM-2, and ZHM-3. (**A**) Colony morphology on PDA medium; (**B**) Mycelium and sporangium; (**C**) Sporangiospore.

Three fungal strains causing mildew were isolated from moldy tobacco leaves and designated as ZHM-1, ZHM-2, and ZHM-3. The results of the morphological observations carried out under a microscope are shown in Fig. 1. The left and right parts of the figure show the culture characteristics of the colonies of each dominant mildew strain and structural characteristics of the fungal body, respectively. Specifically, the morphological characteristics of the three strains were as follows:

ZHM-1: The colonies were loose and spread rapidly, initially white, yellow-green and eventually black. The top sac of the conidial head was spherical and large, the small stem on the spore head was dense, and the spores black and spherical.

ZHM-2: The colonies were loose and grew relatively fast, initially white gradually turning to yellow. The surface of the spore top capsule produced radiating small stalks, small stem monolayer, conidia spherical and rough.

ZHM-3: Hyphae white transparent, no septum, sporulation stem cluster, upright or curved, the base of crestrate filament. Sporangium terminal, spherical or nearly spherical, dark brown. Nostospores were light brown, ovate, spherical or nearly spherical, varying in size.

**Fig. 2:**
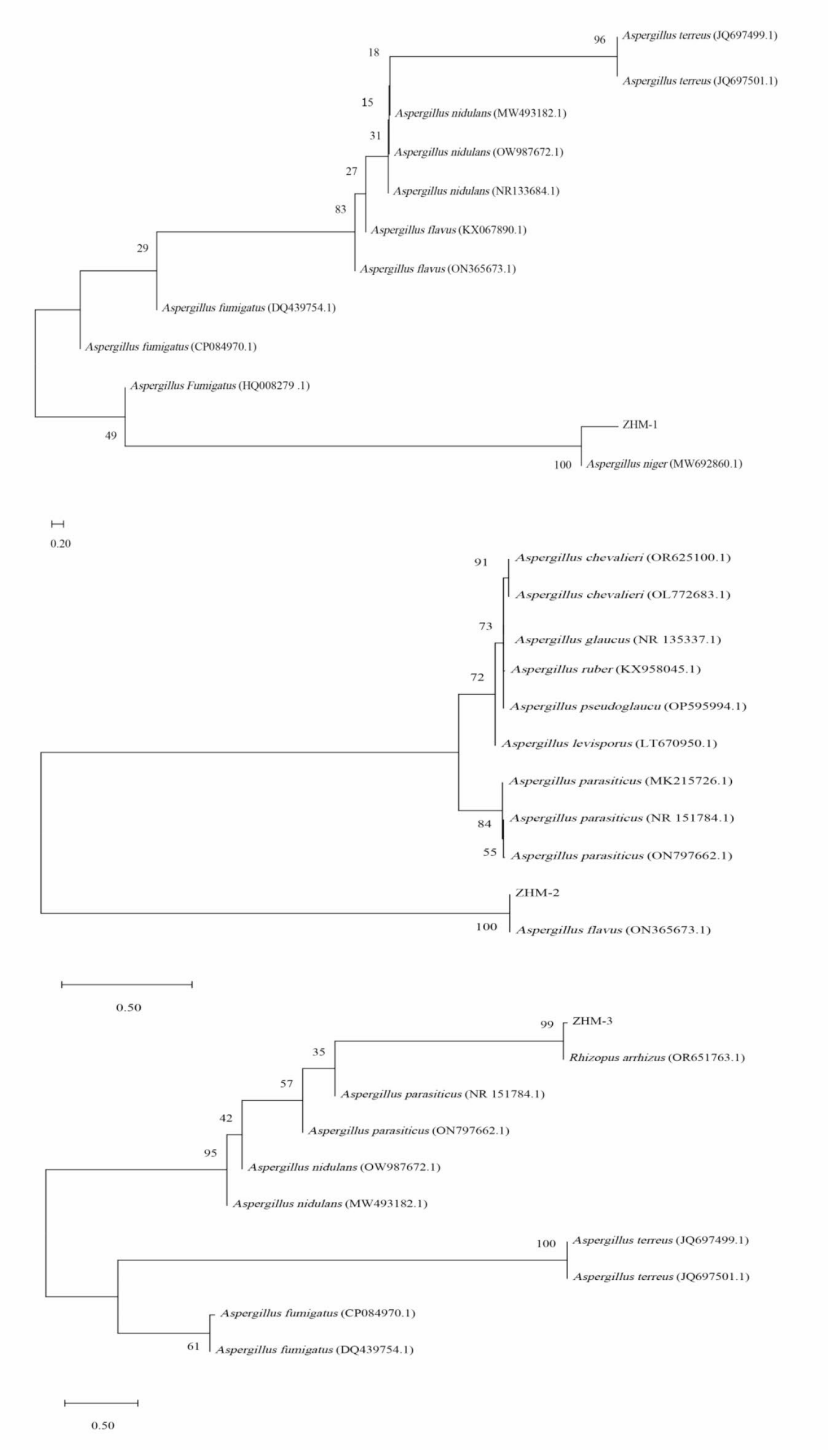
Phylogenetic evolutionary tree of strains ZHM-1, ZHM-2 and ZHM-3.

The ITS sequence PCR products ZHM-1, ZHM-2, and ZHM-3 strains were 570, 573 and 606 bp in size, respectively. The sequences were submitted to GenBank (accession numbers PP818980, PP818981, and PP818982, respectively). BLAST sequence alignment results showed that ZHM-1, ZHM-2, and ZHM-3 shared 99% identity with *A. niger* (KX901281.1), *A. flavus* (PP430395.1) and *Rhizopus arrhizus* (MH715977.1), respectively. The phylogenetic tree of ITS sequences showed ZHM-1, ZHM-2, and ZHM-3 were well matched with the reference sequences of *A. niger* (MW692860.1), *A. flavus* (ON365673.1) and *Rhizopus arrhizus* (OR651763.1) with high bootstrap values 100%, 100% and 99%, respectively.The phylogenetic tree analysis results combined with colony culture characteristics and body structure further confirmed that ZHM-1, ZHM-2, and ZHM-3 were *A. niger*, *A. flavus*, and *R. arrhizus*, respectively (Fig. 2).

### Pathogenicity testing

**Fig. 3:**
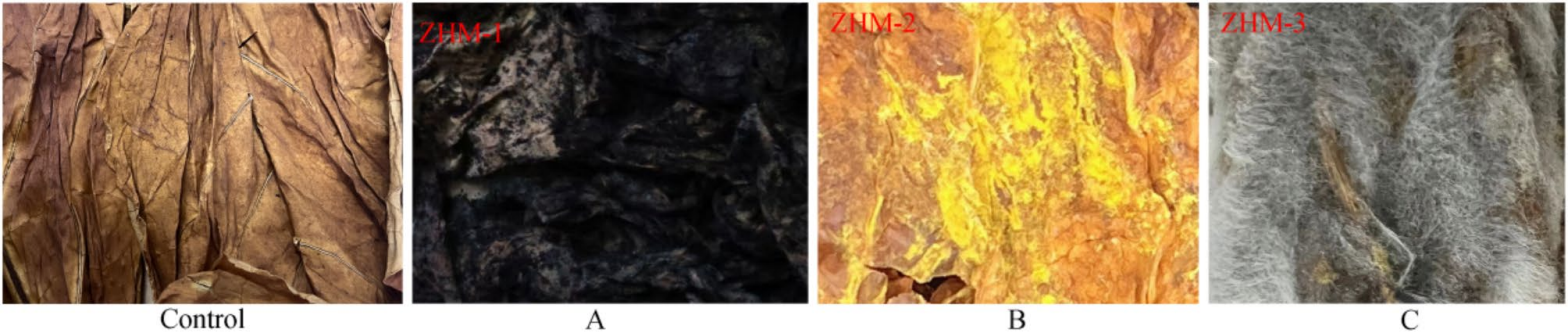
Flue-cured tobacco treated with different mildew spore suspensions. Control. (**A**) treatment 1–1: spraying strain ZHM-1 spore suspension; (**B**) treatment 1–2: spraying strain ZHM-2 spore suspension; (**C**) treatment 1–3: spraying strain ZHM-3 spore suspension.

Flue-cured tobacco leaves treated with different mildew spore suspensions are shown in Fig. 3. Control tobacco leaf inoculated with sterile water did not show any signs of mould.Tobacco leaf surfaces in treatments 1–1, 1–2 and 1–3 were covered with mildew mycelium and had a characteristic mildew odor. All three mildew strains were highly pathogenic; therefore, ZHM-1 (*A. niger*), ZHM-2 (*A. flavus*), and ZHM-3 (*R. arrhizus*) were selected as indicators for screening antagonistic bacterial strains. The re-isolated fungus was identical to the original pure culture.

### Screening and identification of antagonistic bacteria

Primary bacterial screening: In total, 226 bacterial strains were isolated from healthy tobacco samples using the gradient dilution coating method, and five strains of bacteria with antagonistic activity against the five strains of dominant mildew were selected using the plate confrontation method.

**Fig. 4:**
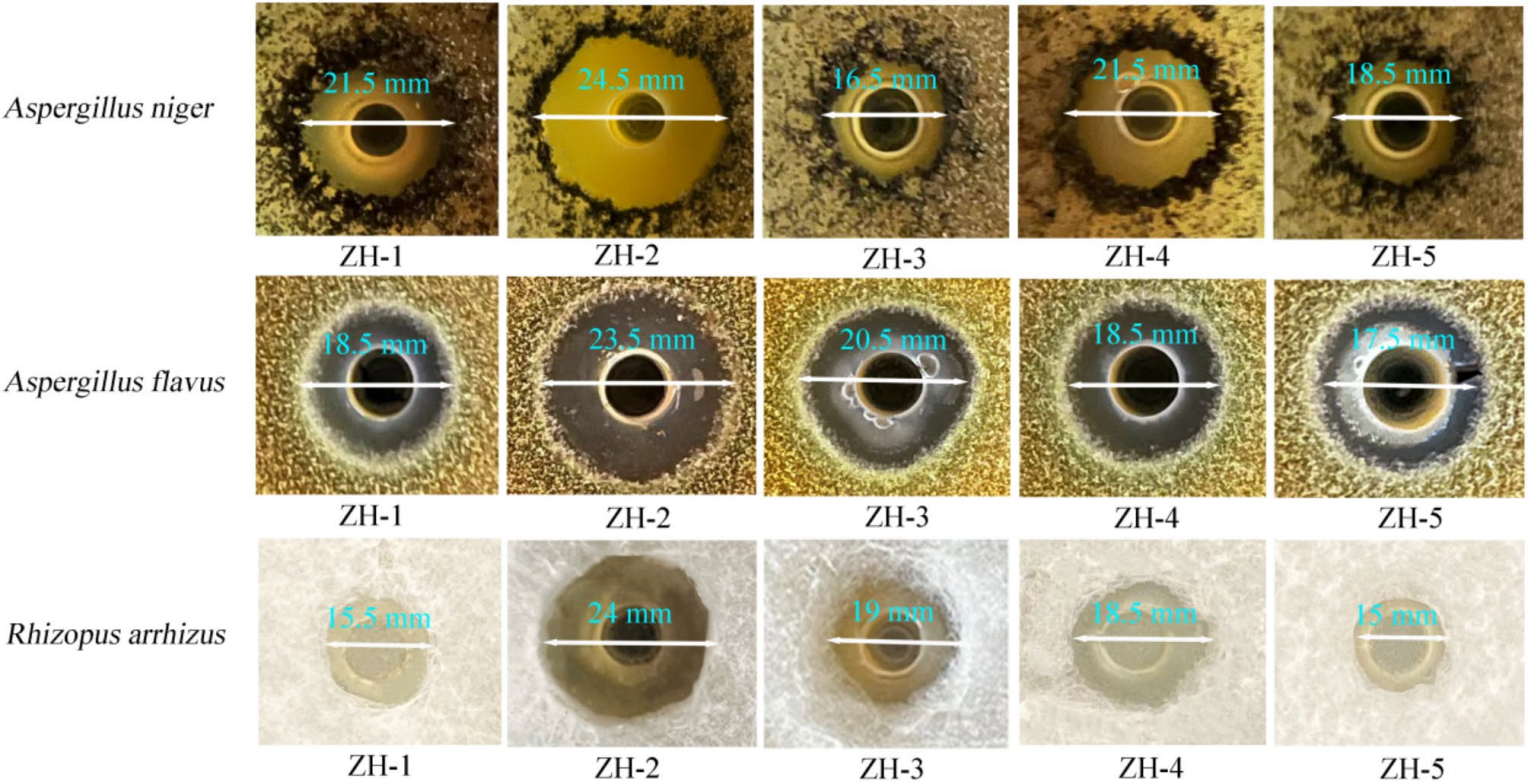
5 antagonistic effects of biobacteria on different mildew.

Rescreening: The results revealed that the fermentation broths of ZH-1, ZH-2, ZH-3, ZH-4, and ZH-5 strains had inhibitory effects on the three mildew strains as indicated by the inhibitory circles of different sizes appearing around the Oxford cups (Fig. 4). The results in Table 1 indicate that the six strains of *Bacillus* microorganisms were more antagonistic to *A. niger* and had a broad-spectrum of antifungal abilities. Among them, ZH-2 had the strongest inhibitory effect on the three mildew strains as indicated by the diameter of the transparent circle which was greater than 22 mm. Therefore, the bacterial strain ZH-2 was selected for subsequent tests.

**Table 1:** The inhibitory effects of different antagonistic bacteria on pathogens.

Species	Mould		
	*Aspergillus niger*	*Aspergillus flavus*	*Rhizopus arrhizus*
ZH-1	++++	+++	++
ZH-2	+++++	+++++	+++++
ZH-3	++	++++	+++
ZH-4	++++	+++	+++
ZH-5	+++	++	+

The data in the table are the mean values of the diameter of the circle of inhibition in three parallel tests, “ -” is no antagonism; “+” is weak antagonism, the diameter of the circle of inhibition is ≤ 15 mm; “++” is medium antagonism, 15 mm < diameter of the circle of inhibition is ≤ 18 mm; “+++” is strong antagonism, 18 mm < diameter of the circle of inhibition is ≤ 20 mm; “++++” is strong antagonism, 20 mm < diameter of the circle of inhibition is ≤ 22 mm; “+++++” is the strongest antagonism, the diameter of the circle of inhibition is > 22 mm.

**Fig. 5:**
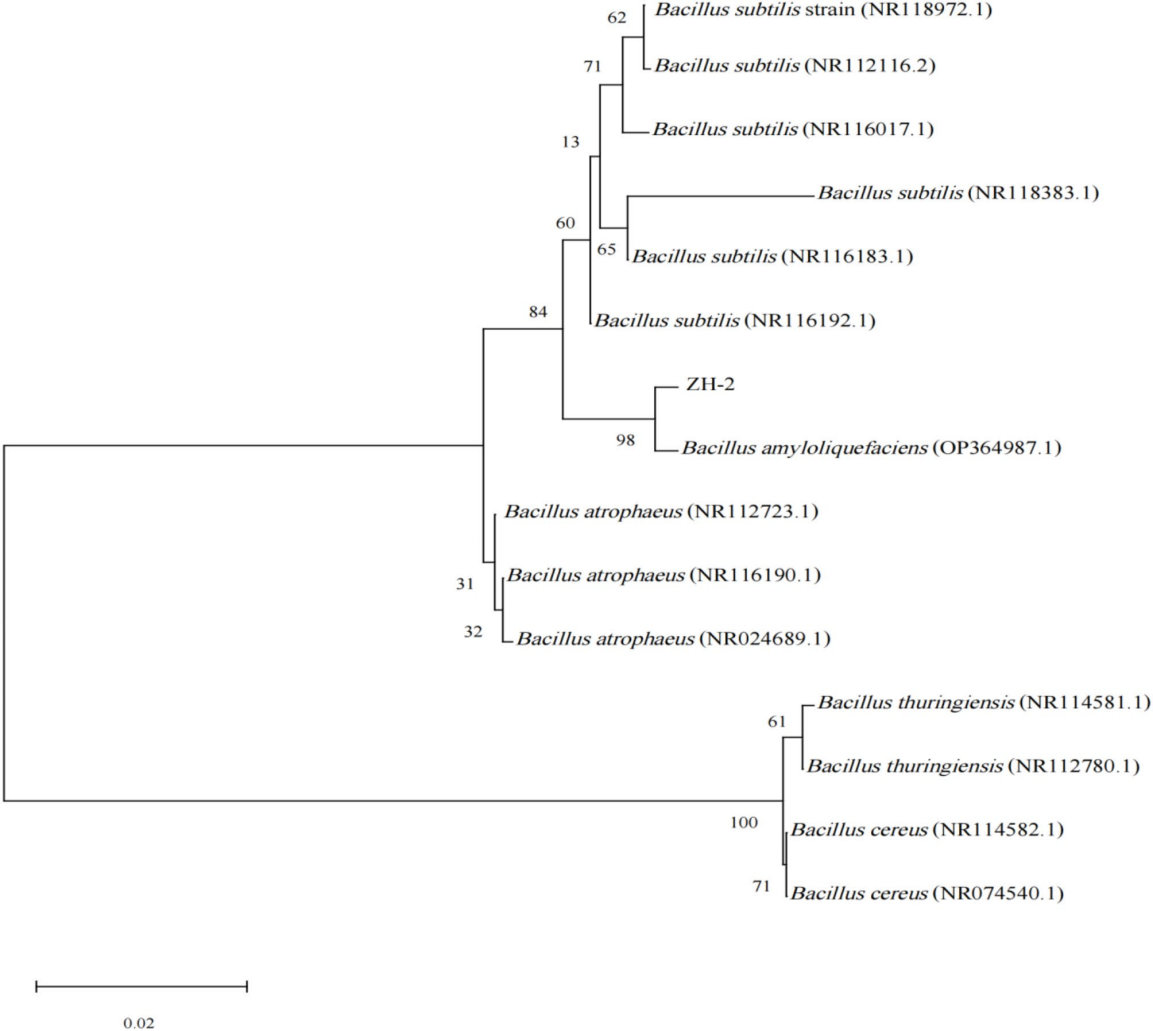
A phylogenetic evolutionary tree of strain ZH-2.

A 1455 bp PCR product of bacterial strain ZH-2 (GenBank Accession No. PP800225) was obtained and sequenced. Homology analysis using the NCBI BLAST revealed that the 16 S rDNA gene sequence of strain ZH-2 showed a 99% similarity to *B. amyloliquefaciens* (PP236930.1). In addition, the phylogenetic tree of 16 S sequences showed ZH-2 was well matched with the reference sequences of *B. amyloliquefaciens* (OP364987.1) with a high bootstrap value (98%) (Fig. 5). Therefore, the strain was identified as *B. amyloliquefaciens* ZH-2 based on NCBI BLAST and phylogenetic analyses.

### Biochemical characteristics of the ZH-2 strain

As shown in Table 2, strain ZH-2 is a gram-positive bacterium and elicited positive reactions in V-P determination, nitrate reduction, gelatin liquefaction, starch hydrolysis, and catalase tests. In contrast, citrate utilization, methyl red, and hydrogen sulfide tests of strain ZH-2 were negative.

**Table 2:** Biochemical characteristics of strain ZH-2.

Index	Characteristics
Gram staining test	+
V-P determination test	+
Citrate utilization test	−
Nitrate reduction test	+
Gelatin liquefaction test	+
Methyl red test	−
Starch hydrolysis test	+
Hydrogen sulfide test	−
Catalase test	+

+: Positive reaction; −: Negative reaction.

### Scanning electron microscopy observation of pathogenic bacteria

**Fig. 6:**
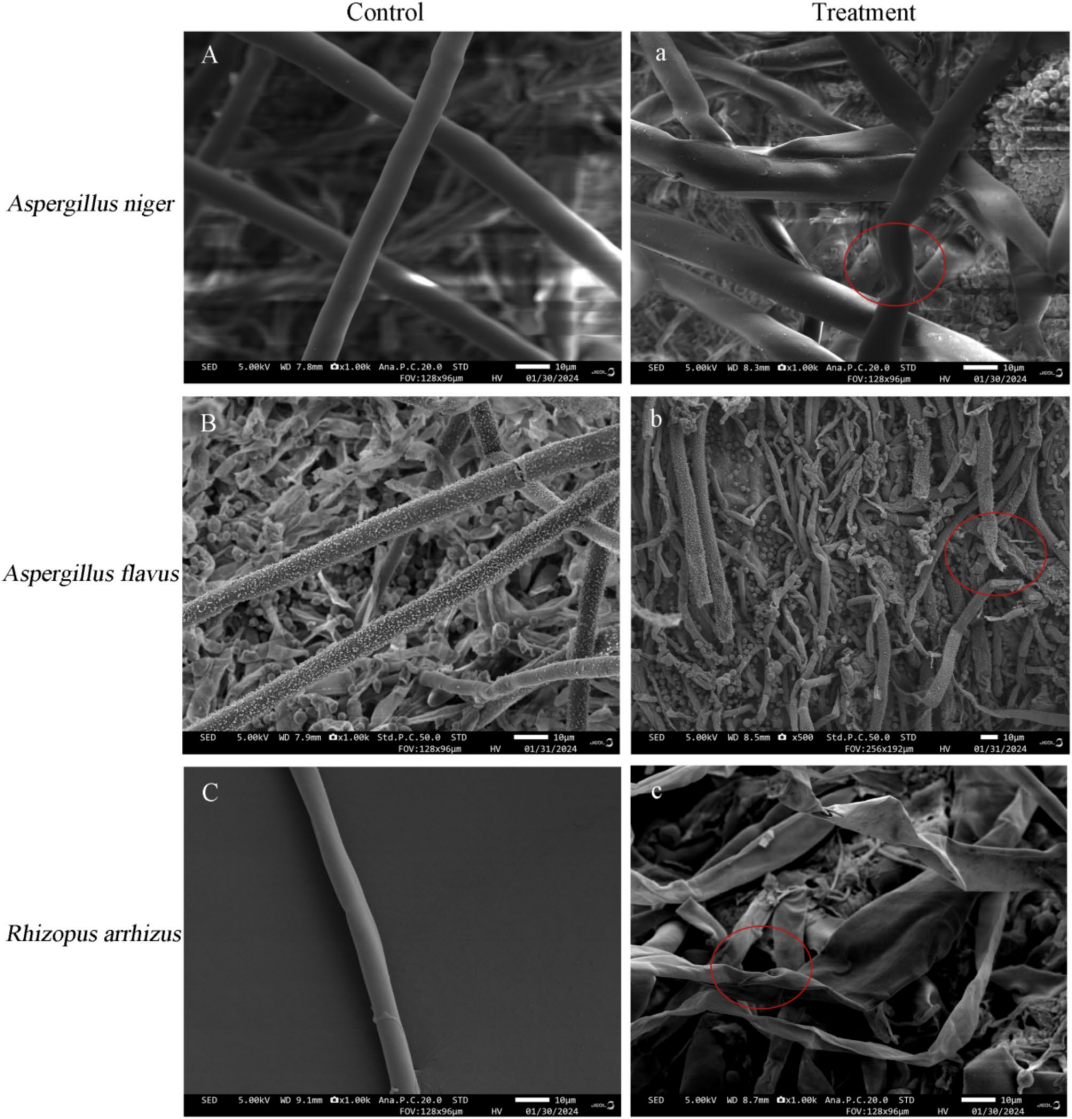
Inhibition effect of strain ZH-2 on the mycelial growth of three pathogenic fungi. (**A**–**C**) are control groups treated with sterile water; (**a**–**c**) are treatment groups with the addition of strain ZH-2, and the red oval frame in (**a**–**c**) points to three pathogenic fungi mycelium is significantly damaged.

Scanning electron microscopy was used to observe the three dominant mildew strains and pathogenic bacteria infested by the ZH-2 antagonist bacterial strain (Fig. 6).

Figure 6A shows the normal growth of *A. niger* mycelia, characterized by a dense and smooth mycelial surface and full and uniform shape. Infestation with the ZH-2 strain resulted in *A. niger* mycelia that were twisted and deformed, shrivel-concave and dried up with a rough and uneven mycelial surface (Fig. 6a).

Figure 6B shows the normal growth of *A. flavus*, characterized by round and full mycelia, and a complete morphological structure. It also shows *A. flavus* mycelia infested by strain ZH-2, which were broken, severely atrophied, flaked, irregularly folded, and ulcerated, and characterized by a deformed appearance (Fig. 6b).

Figure 6C shows the normal growth of mycelia of *R. arrhizus*, characterized by uniform thickness and smoothness. After infestation with strain ZH-2, mycelia of *R. arrhizus* became rough and signs of twisting, crumpling, and clustering, and an increase in abnormal branching appeared (Fig. 6c).

### Production of extracellular cell wall degrading enzymes by strain ZH-2

**Fig. 7:**
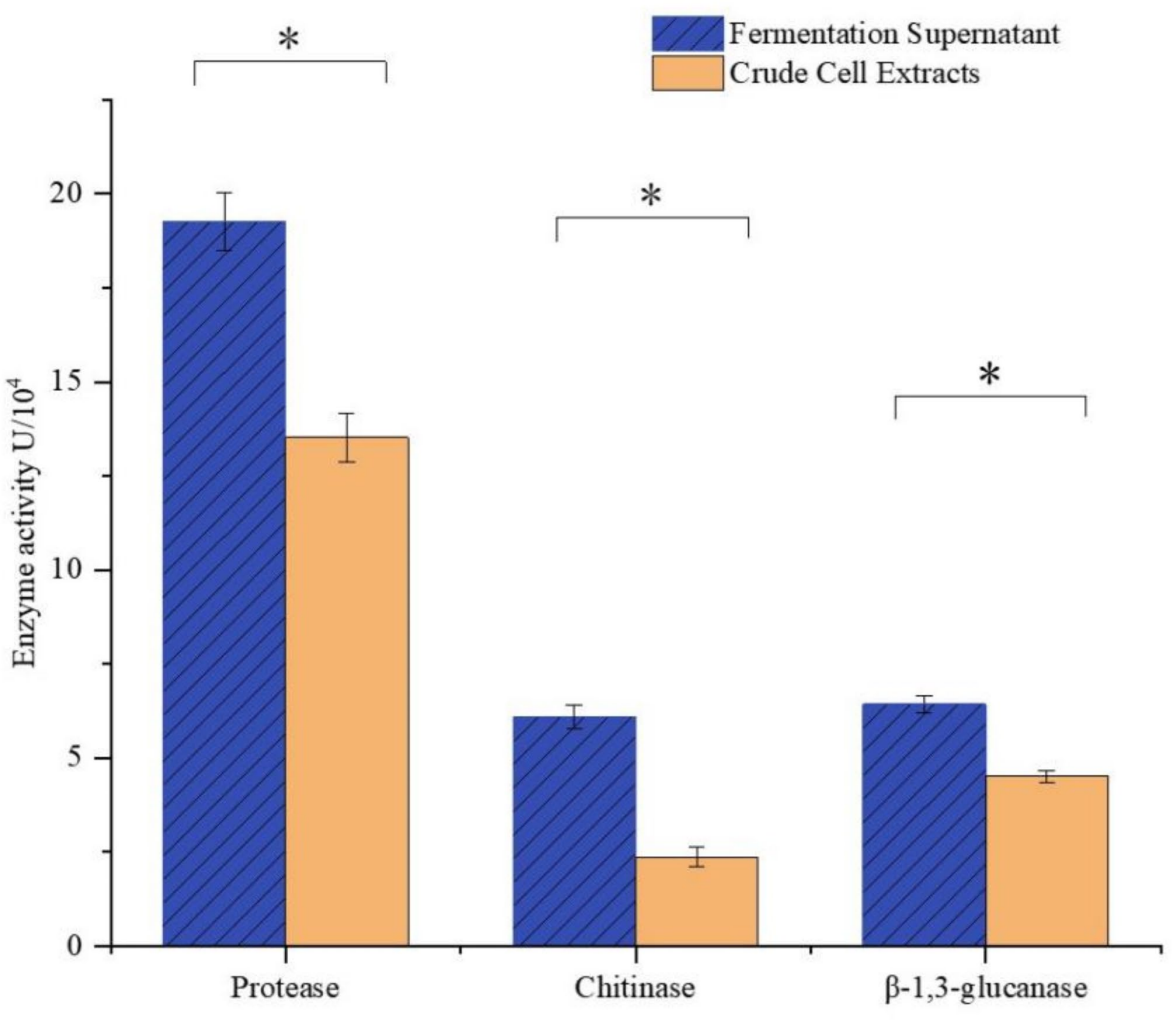
The activity of fungal cell wall hydrolytic enzymes in the Fermentation Supernatant and Crude Cell Extracts of *Bacillus amyloliquefaciens* ZH-2. The error bars represent the standard errors of the means calculated from at least three replicates for each treatment. Asterisks (*) represent signification differences between Fermentation Supernatant and Crude Cell Extracts according to independent sample t-test (*P* < 0.05).

The ability of ZH-2 to produce extracellular cell wall–degrading enzymes was investigated to investigate its antagonistic mechanisms. Protease, chitinase, and β−1,3-glucanase were detected in the fermentation supernatant and in the cellular crude extracts of strain ZH-2. Both enzymes were detected in significantly higher levels in the supernatant than in cellular crude extract phase (Fig. 7). This indicates that the strain can produce protease, chitinase, and β−1,3-glucanase to break down the cell wall of pathogenic bacteria.

### Effect of antagonistic strain ZH-2 on mildew prevention of flue-cured tobacco

**Fig. 8:**
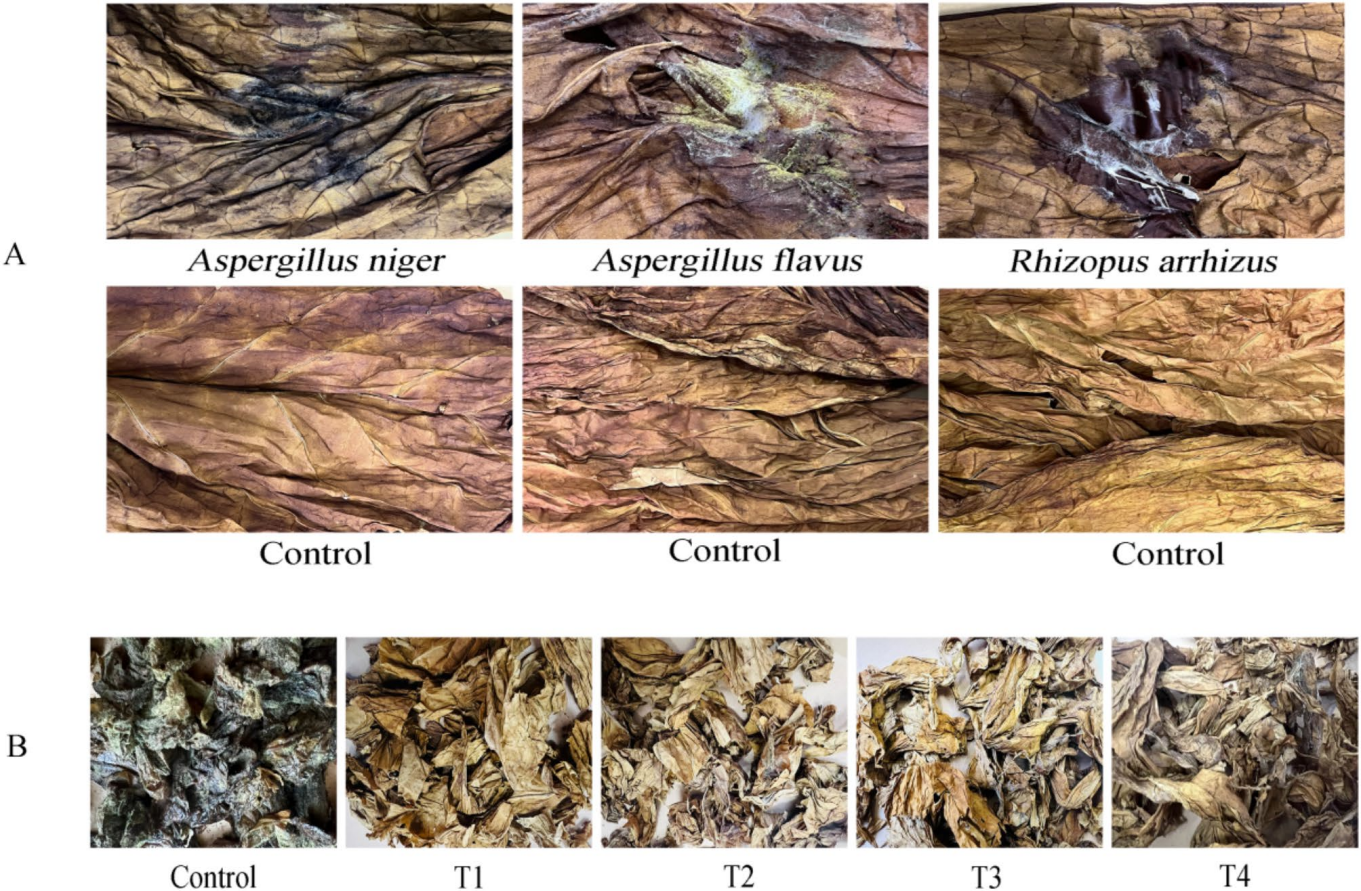
The mildew control effect of ZH-2 bacterial suspension in tobacco leaves. (**A**) Efficacy against a single pathogen. (**B**) Control effect on mixed mold.

The control effect of strain ZH-2 on single pathogen is shown in Fig. 8A. The results showed that the tobacco leaf was corrupted and lost luster after 15 days. However, there was no obvious mildew on the tobacco leaf surface of the control group.Different concentrations of the ZH-2 bacterial suspension had a significant inhibitory effect on the growth of mildew and the effect increased significantly with increasing concentration (Fig. 8B). After 7 d, the CK surface inoculated with a mixed bacterial solution was covered with abundant mildew (tobacco rot) in flue-cured tobacco leaves. As the concentration of bacterial suspension in treatments T1–T4 increased, tobacco mildew disease severity progressively decreased. As shown in Table 3, the disease incidence rate of tobacco leaves in treatments T1–T4 ranged between 13.33 ~ 61.11% and the disease between 5.93 ~ 45.56%, and the biocontrol efficacy reached 46.52–93.04%. The disease incidence and disease index were significantly reduced following inoculation with the bacterial suspensions (*P* < 0.05). The relative control effect was T1 > T2 > T3 > T4, implying that the control effect on tobacco mildew disease decreased as the concentration of the bacterial suspension decreased. These results indicate that strain ZH-2 significantly reduces the occurrence of tobacco mildew. The higher its concentration is, the better is its relative prevention effect.

**Table 3:** Prevention and control effect of *Bacillus amyloliquefaciens* ZH-2 on mildew in tobacco leaves.

Treatment	Morbidity/%	Disease/%	Relative/%
CK	100.00 ± 0.00^e^	85.19 ± 1.49^e^	
T1	13.33 ± 3.34^d^	5.93 ± 0.97^d^	93.04
T2	27.78 ± 3.85^c^	15.31 ± 2.99^c^	82.03
T3	45.55 ± 3.85^b^	28.64 ± 1.13^b^	66.38
T4	61.11 ± 1.92^a^	45.56 ± 0.38^a^	46.52

Data are presented as means of three replicates 3 standard deviations. Different lower-case letters in each column indicates that means are significantly different by the LSD test (*p* < 0.05).

### Analysis of the conventional chemical composition of flue-cured tobacco

**Fig. 9:**
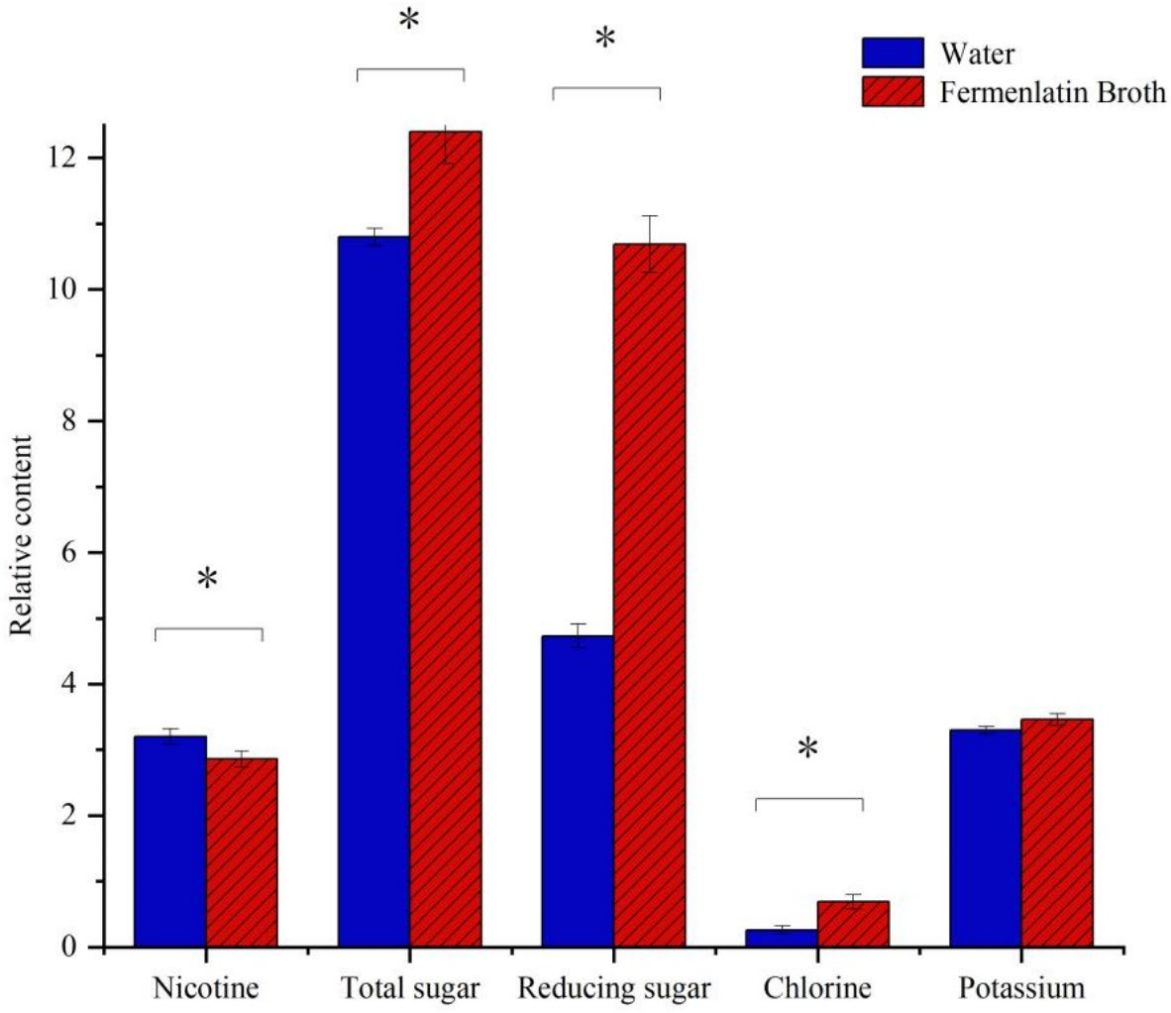
Effect of fermentation broth treatment on the conventional chemical composition content of tobacco leaves. Error bars represent standard error of the mean. Statistical significance was determined according to independent sample t-test and asterisks (*) indicates significant differences at *P* < 0.05.

The conventional chemical composition of flue-cured tobacco leaves includes nicotine, total sugar, reducing sugar, chlorine, and potassium. The contents of these substances are closely related to the tobacco quality. The nicotine content of fermented tobacco was 2.86%, which is significantly smaller (by 10.62%) than that of the freshwater treatment. In contrast, the total and reducing sugar contents were 12.4% and 10.69%, respectively. They were significantly higher (by 14.8% and 126%, respectively) than those of the freshwater treatment. In addition, the potassium content in the treated flue-cured tobacco increased; the chloride content increased but remained within the appropriate range (Fig. 9).

### Degradation effect of macromolecular substances

**Fig. 10:**
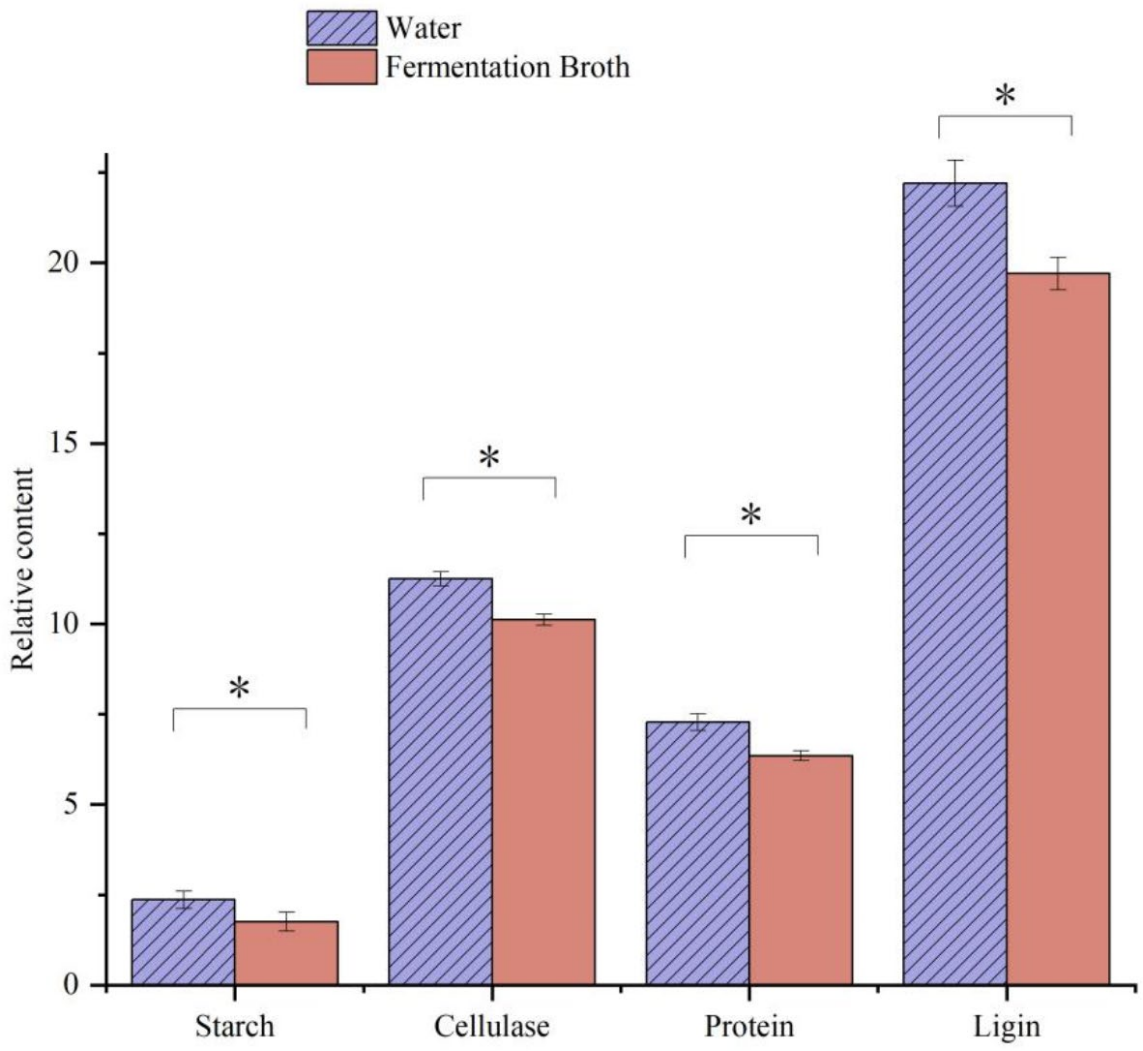
Effect of fermentation broth treatment on macromolecular material content in tobacco leaves. Error bars represent standard error of the mean. Statistical significance was determined according to independent sample t-test and asterisks (*) indicates significant differences at *P* < 0.05.

Macromolecular substances in tobacco leaves with high content that have a larger impact on the tobacco quality include starch, cellulose, lignin, and protein. After fermentation, the starch content of tobacco was 1.75%, which was 25.85% lower than that of sprayed water; the protein content was 6.35%, that is, 12.77% lower than that of sprayed water; the cellulose content was 10.12%, which was 10.04% lower than that of sprayed water; and the lignin content was 19.7%, which was 11.26% lower than that of sprayed water (Fig. 10).

### Variation in the volatile flavor compounds

**Fig. 11:**
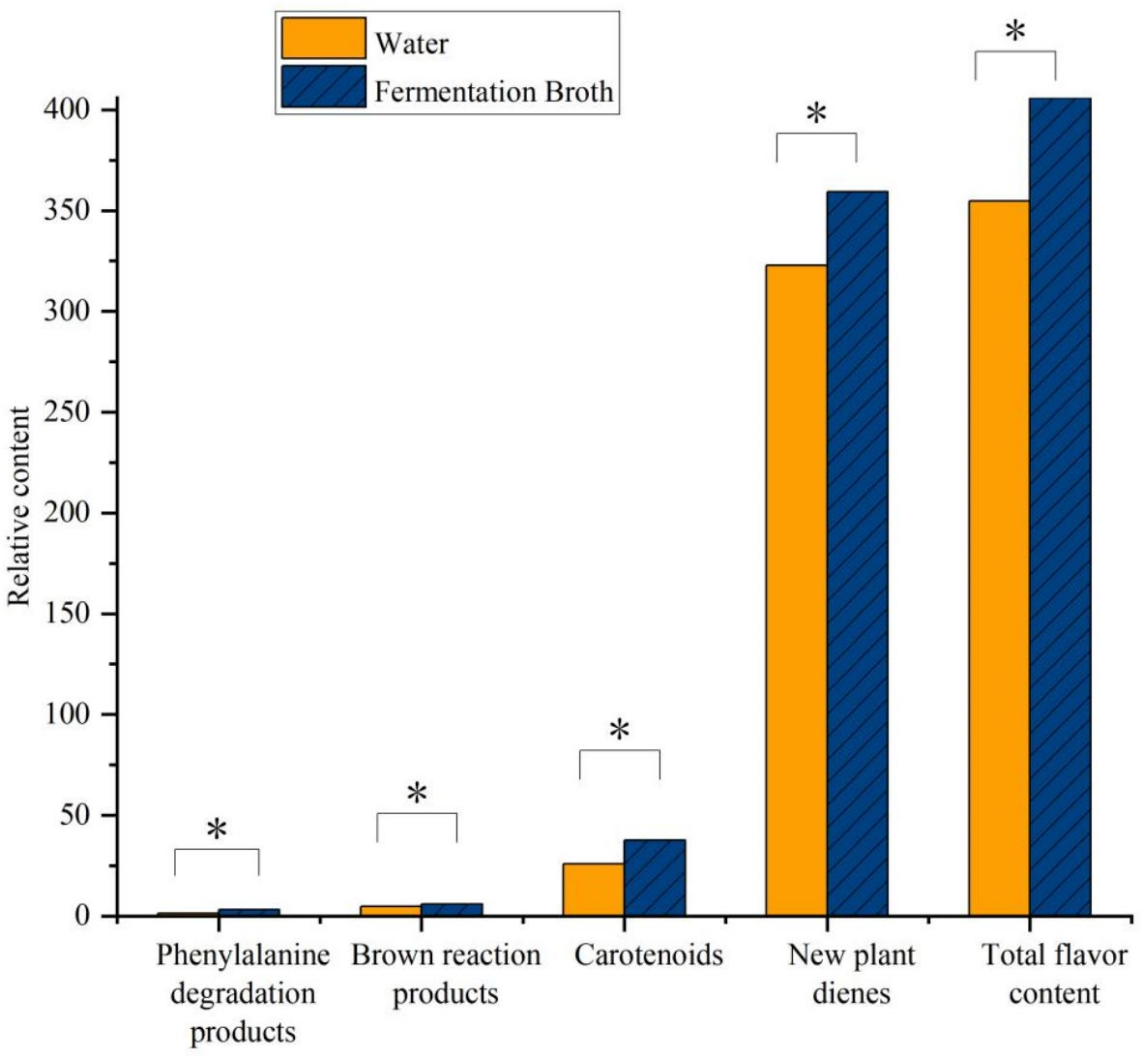
Effect of fermentation broth treatment on volatile flavor compounds in tobacco leaves. Error bars represent standard error of the mean. Statistical significance was determined according to independent sample t-test and asterisks (*) indicates significant differences at *P* < 0.05.

In the different treatments, 29 volatile flavor substances were identified. They can be divided into four categories: phenylalanine degradation products, brown reaction products, carotenoids, and new plant dienes. Figure 11 shows that the total flavor content increased after fermentation. The contents of four types of flavor substances increased compared with that of the clean water treatment (alanine degradation products, by 121.58%; brown reaction products, by 24.62%; carotenoids, by 44.80%; and new dienes, by 11.37%). These results show that ZH-2 further improved the tobacco leaf quality.

## Discussion

Tobacco mildew is a common fungal disease that causes leaf rot during tobacco storage. It seriously impacts the yield and quality of tobacco and is of great economic importance. Therefore, an in-depth investigation of the inhibitory effect of endophytic bacteria on fungal pathogens is of great significance for tobacco mildew biocontrol. In this study, three dominant pathogens were isolated from tobacco mildew tissues and identified as *A. niger*, *A. flavus*, and *R. arrhizus* using morphological observation, pathogenicity tests, and multi-gene sequence analysis. *A. niger* and *A. flavus*cause mildew spoilage of produce, fruits, and vegetables with high moisture content^32,33^. *R. arrhizus*is a widely distributed pathogen because of its wide range of hosts, rapid growth rate under suitable conditions, and secretion of cell wall hydrolytic enzymes^34,35^. Reports on tobacco mildew biological control are limited. Kortekamp^16^ reported that *Pseudomonas fluorescens* can protect against the the natural infection of flue-cured tobacco fungi based on an in vitro assay.

In this study, five strains with strong antagonistic effects against the three dominant mildews were isolated from healthy tobacco leaves after screening. *B. amyloliquefaciens* strain ZH-2 exhibited the highest inhibitory action as indicated by the diameter of the inhibition circle exceeded 22 mm. In addition, different genera causing mildew were used in the test, which indicates that the selected strains had a broader spectrum of inhibitory effects. Thus, it is possible that they can also effectively inhibit other mildew-causing fungi besides the dominant mildew tested in the flue-cured tobacco. The strong resistance of *Bacillus amyloliquefaciens*may be attributed to its ability to inhibit mycelial growth and spore germination and to produce lipopeptide antibiotics, bacteriostatic proteins, and other substances^36–38^. In this study, the inhibitory effect of the bacterial isolates was further confirmed by scanning electron microscopy of mycelia in the inhibition zone. Mycelia co-cultured with antagonistic strains showed fractures, atrophy, and ulceration. This agrees with previous results based on which *Bacillus *KTMA4 successfully inhibits plant pathogens and causes mycelium deformation and shrinkage^39^. *A. niger*, *(A) flavus*, and *R. arrhizus*are common fungi. The main components of the fungal cell wall are chitinase, β−1,3-glucanase, and protease^40,41^. Secretion of extracellular hydrolases is one of the most important biological control mechanisms of antagonistic strains^42–44^. The results of this study showed that *(B) amyloliquefaciens* ZH-2 strain’s both fermentation supernatant and cell crude extracts had a high chitinase, β−1,3-glucanase, and protease content. It was hypothesized that *B. amyloliquefaciens*ZH-2 affects the enzymes involved in the synthesis of the cell wall based on the production of these hydrolytic enzymes, leading to morphological changes in the fungal hyphae, cell wall degradation, mycelium leakage, and ultimately to apoptosis. Furthermore, multiple cell wall hydrolytic enzymes produced by the ZH-2 strain could have synergistic antifungal activities. Based on previous studies, a combination of cell wall hydrolytic enzymes, chitinase, β−1,3-glucanase, and protease produces synergistic antifungal effects. It accelerates cell wall degradation and improves biocontrol effect compared with the action of a single enzyme.This can be attributed to the fact that because cell wall chitin and β-glucan polymers are crosslinked and embedded in the glycoprotein polymer^45–47^. And the degradation of any one of the fungal cell wall components increases the exposure of the other structural polymers^46,47^. Thus, a combination of these cell wall hydrolytic enzymes produced by ZH-2 can accelerate the degradation and deformation of fungal cell walls and increase its antifungal activity. The production of cell wall hydrolases is a possible mechanism of antifungal action of ZH-2 against the three dominant fungi in this study. This mechanism has been identified in previous studies to be an effective mechanism for the inhibition of pathogens^48,50^. For example, Ayub et al.^51^ reported that *Rhizobium* and *Burkholderia* are able to control the pathogenic fungus *Fusarium oxysporum* by producing cell wall hydrolytic enzymes with an antifungal action. Kaur et al.^52^ reported similar results in their study on the interaction between *Streptomyces spp*. and *Bacillus sp*. R2. They observed that hyphae shrank, collapsed, empty hyphae, and had large depressions and a loss of turgidness. The mode of antifungal action of *Bacillus subtilis*BS-58 against two destructive plant pathogens was the production of antifungal metabolites^53^. The results of this study demonstrate that antifungal metabolites are extracellular in nature and inhibit fungal growth by diffusion into the culture medium. The efficacy of the present study in controlling tobacco mildew disease in a laboratory setting compared with the control demonstrates the potential of *Bacillus amyloliquefaciens* ZH-2 as an efficient biocontrol resource.

Furthermore, this study showed that spraying with *B. amyloliquefaciens* ZH-2 improves the quality of flue-cured tobacco leaves. Tobacco fermentation is an important process for improving tobacco quality. This process can be accelerated by inoculating tobacco with microorganisms^54^. Wen et al.^55^ and Ma et al.^56^ screened *Bacillus amyloliquefaciens* and *Bacillus subtilis* strains for the solid-state fermentation of tobacco leaves, which degraded starch and protein, improved the chemical quality and flavor, promoted the production of flavor substances, improved the quality of tobacco leaves, and had good application prospects. In this study, the chemical composition of conventional tobacco was determined after spraying it with the fermentation broth of the ZH-2 strain. The alkaloid content significantly decreased (by 10.62%) compared with that of the control, which reduced the potential threat of alkaloids and improved the safety. In addition, the total and reducing sugar contents were significantly higher than those of the control group (by 12.9% and 55.75%, respectively), which significantly increased the total and reducing sugar contents in the fermented tobacco, reduced irritation, and improved the aroma and taste. In this study, the contents of starch, protein, lignin and cellulose were significantly lower than those of the control, which led to fine smoke, weakened irritation and miscellaneous gas, and improved flavor quality. Phenylalanine degradation products, brown reaction products, carotenoids, and new plant diene are the main flavoring compounds in tobacco^57,58^. Our results show that the content of the four flavoring substances of tobacco increased after fermentation, leading to enhanced smoke flavor, improved sucking flavor, toning down of smoke, and reduced irritation, all of which enhanced tobacco quality.

## Conclusion

In this study, three strains of dominant tobacco mildew pathogens, *A. niger*, *(A) flavus*, and *R. arrhizus*, were isolated from the surface of diseased tobacco leaves and used as pathogens to select five antagonistic bacterial strains with good inhibitory effects against tobacco mildew disease. The most effective broad-spectrum antagonist strain, *(B) amyloliquefaciens* ZH-2, was selected for subsequent tests. The results revealed that the antagonistic mechanism of ZH-2 against pathogens was the production of cell wall hydrolases, such as chitinase, β−1,3 glucanase, and protease, which destroy the mycelial cell wall and ultimately inhibit the growth of fungi. Fermentation results showed that strain ZH-2 affected the chemical composition and improved the volatile flavor content and quality of tobacco leaves. Therefore, strain ZH-2 can be used as a potential biocontrol agent for the control of the tobacco mildew disease and the improvement of tobacco quality during storage. In conclusion, this study provided crucial insights and identified bacterial biocontrol resources for controlling tobacco mildew control and improving tobacco quality.

## Data Availability

All relevant data are reported in the article. Additional data can be provided upon request by the corresponding author.
